# Supplementary material for: Embedding the value of coastal ecosystem services into climate change adaptation planning
Source: PeerJ. 2022 Aug 23;10:e13463. doi: 10.7717/peerj.13463 (PMC9415443; doi:10.7717/peerj.13463)
Supplement: Supplemental Information 1 [file peerj-10-13463-s001.docx]

**Supplemental Material for Wedding et al. (2022) Embedding the value of coastal ecosystem services into climate change adaptation planning. *Peer J***

We used the Integrated Valuation of Ecosystem Services and Tradeoffs (InVEST) Coastal Vulnerability model to investigate where habitats protect against the impacts of coastal hazards and translate these results to support adaptation planning.

**GIS Data Inputs**

The InVEST Coastal Vulnerability model produces a qualitative estimate of coastal exposure in terms of an Exposure Index, which differentiates areas with relatively high or low exposure to erosion and inundation during storms (Table S1). Model inputs, which serve as proxies for various complex shoreline processes that influence exposure to erosion and inundation, include:

Table S1. List of variables and ranking system for inputs to Coastal Vulnerability model. Relative risk of erosion and inundation is scaled from 1 to 5.

| Risk category | Very Low | Low | Moderate | High | Very High |
| --- | --- | --- | --- | --- | --- |
| Numeric rank | **1** | **2** | **3** | **4** | **5** |
| Geomorphology | Rocky shore, high cliffs, seawalls | Medium cliff, sheltered coast, bulkheads, small seawalls | Low cliff, glacial drift, alluvial plain, revetments, rip-rap | Cobble beach, estuary, lagoon, bluff | Barrier beach, sand beach, mud flat, delta |
| Neighbor effect of seawalls (ranking change) |  |  | Non-sand, non-mud geomorphology next to seawall | Mud geomorphology next to seawall | Sand geomorphology next to seawall |
| Relief | 81^st^ – 100^th^ percentile | 61^st^ – 80^th^ percentile | 41^st^ – 60^th^ percentile | 21^st^ – 40^th^ percentile | 0^th^ – 20^th^ percentile |
| Natural Habitats |  | High sand dune, wetland | Low sand dune | Seagrass, kelp | No habitat |
| Sea Level Change | 0^th^ – 20^th^ percentile | 21^st^ – 40^th^ percentile | 41^st^ – 60^th^ percentile | 61^st^ – 80^th^ percentile | 81^st^ – 100^th^ percentile |
| Wave Exposure | 0^th^ – 20^th^ percentile | 21^st^ – 40^th^ percentile | 41^st^ – 60^th^ percentile | 61^st^ – 80^th^ percentile | 81^st^ – 100^th^ percentile |
| Wind Exposure | 0^th^ – 20^th^ percentile | 21^st^ – 40^th^ percentile | 41^st^ – 60^th^ percentile | 61^st^ – 80^th^ percentile | 81^st^ – 100^th^ percentile |
| Surge Potential | 0^th^ – 20^th^ percentile | 21^st^ – 40^th^ percentile | 41^st^ – 60^th^ percentile | 61^st^ – 80^th^ percentile | 81^st^ – 100^th^ percentile |

1. **Geomorphology:** A polyline with attributes about local coastal geomorphology along the shoreline based on the NOAA [Environmental Sensitivity Index](https://coast.noaa.gov/digitalcoast/data/esi.html) (National Oceanic and Atmospheric Administration, Office of Response and Restoration, 2002). This line shapefile input is used to compute the Geomorphology ranking (1-5) of each shoreline segment. For example, rocky cliffs (Rank=1) are less prone to erosion and inundation than estuaries (Rank=4) or sandy beaches (Rank=5). The model does not account for predicted changes in the coastline or its attributes due to sea level rise, erosion, or changes to sediment dynamics.
2. **Coastal habitat:** Polygons representing the location of natural habitats (e.g., seagrass, kelp, wetlands, etc.) from the California Department of Fish and Wildlife (CDFW) website created for Marine Life Protection Act process (California Department of Fish and Wildlife, 2015). The model uses these input layers to compute a Natural Habitat ranking for each shoreline segment (Table S1). The model does not account for spatial variation in quality, height, or density of vegetation within habitats. When multiple habitats are present within a specified protective distance of a shoreline segment (Table S2), their aggregate protective rank is determined by the following equation, where *R_Hab_* is the ranking of the aggregate habitat (Sharp et al., 2018):

*N*

*N*

$$R_{Habitat}=4.8-0.5\sqrt{(1.5{(5-R_{k}))}^{2}+\sum_{k=1}^{N} {(5-R_{k})}^{2}-{(5-R_{k}) )}^{2}}$$

**Table S2.** Natural habitats confer protection to the coastline in the form of wave attenuation and reduced flooding. Benefits to the coastline depend on distance to that habitat and its position relative to oncoming waves. The protected distances were assigned through iterative discussions with a range of local stakeholders and literature review.

| Habitat | Protective Distance (m) | Habitat Rank |
| --- | --- | --- |
| Kelp | 200 | 4 |
| Low Dune | 300 | 3 |
| High Dune | 300 | 2 |
| Seagrass | 500 | 4 |
| Wetlands | 500 | 3 |

- 1. **Wind and wave exposure:** A point shapefile containing values of observed storm wind speed and wave power across an area of interest (AOI) using Wave Watch III data provided by NOAA. Wind and wave parameters are provided in a grid of points spaced approximately 50 km apart off the coast of Marin County, California (Tolman and National Oceanic and Atmospheric Administration, 2014). For each point, the risk ranking is based on the averaged top 10 percent of recorded values for wind speed and wave height at each of 16 angular headings (0^o^, 22.5^o^, 45^o^_,_ etc.), as opposed to the most extreme values observed at each heading. Wave Watch III does not quantitatively predict how wave parameters during extreme storm events will change in the future. Sheltered coastline segments are less likely to have enough distance of water in front of them for buildup of oceanic waves. Instead the wave height is primarily dependent on local wind-driven waves.

1. **Surge potential:** A polyline of the edge of the continental shelf serves as a proxy for oceanic surge potential. In general, a longer the distance between the coastline and the edge of the continental shelf will result in a higher storm surge. The model does not account for land barriers in front of coastal segments that would alter storm surge. Fetch distance and the amount of shallow water in front of a coastal segment determine the risk of storm surge-related hazards. Areas of shallow water (“sheltered” segments”) have greater potential for surge waves to develop. In the Coastal Vulnerability Model, designations of “sheltered” versus “exposed” segments are categorical. Because the definition of sheltered is relative to other areas of the coastline in the study area, users must specify an approximate proportion of the coast to designate as sheltered as a starting point for the model to identify sheltered segments. (Note that this is not the final proportion of sheltered segments identified in the model.) We designated 20 percent of the coast (sheltered proportion = 0.2) as a starting point for sheltered segments. Maximum fetch distance was 12,000m. The depth threshold for shallow water was ≤ 20m.
2. **Relief:** A 5-meter resolution bathymetry/topography digital elevation model of California’s coastal land and waters from the United States Geological Service (Foxgrover and Barnard, 2012).
3. **Sea-level rise:** The mean sea level datum was 0m. Rates of projected net sea level change through 2030 were derived from local variation in global sea level rise and coastal land subsidence/uplift rates (National Research Council, 2012). We chose the upper range of projections for the San Francisco region for 2030 (29.7 cm of increase) because, based on discussions with Marin County, 2030 is firmly within the planning timeline for the Local Coastal Program updates. They preferred to use the upper range of sea level rise projections to maintain a degree of precaution and these values are comparable with mid-range projections for 2050.

**Exposure Index**


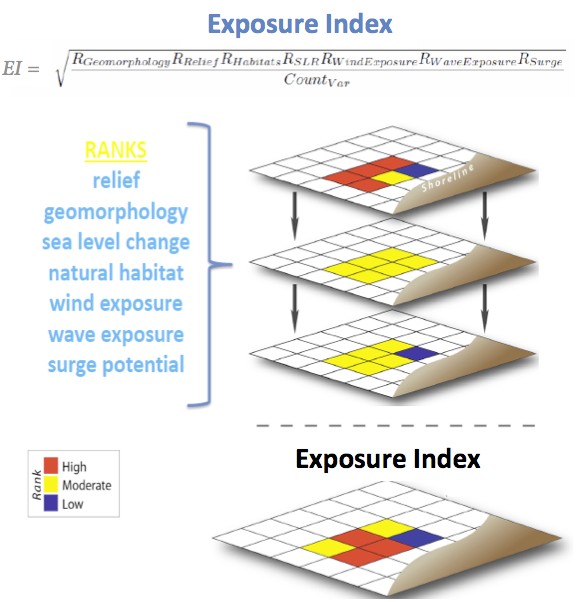


**RANKS**

$EI={(R_{Geomorphology}R_{Relief}R_{Habitats}R_{SeaLevelRise}R_{WindExposure}R_{WaveExposure}R_{Surge})}^{1/7}$

Fig. S1. Schematic of spatially explicit Exposure Index calculation in the InVEST Coastal Vulnerability Model.

We calculated the role of habitats in reducing coastal exposure for every segment of coastline using the difference between two modeled scenarios: one with present day natural habitats (*R_Habitat_* follows rankings in Table S1) and one where all natural habitats were absent (*R_Habitat_ =* 5).

$$Role of habitat= {EI}_{HabitatAbsent}-{EI}_{HabitatPresent}$$

We classified the role of habitat for each segment as Highest, Medium, or Lowest using the Natural Breaks (Jenks) method in ArcMap 10.1. These classifications are relative to the overall distribution of EI reductions and are thus sensitive to area modeled (i.e. a segment with a High value when modeling only Marin County may not be classified as High if considering the entire California coast). While the raw EI values and the role of habitat remain the same when using the same input parameters, the classifications of relative habitat roles that we present are specific to Marin County only.

**Model limitations**

One limitation of this modeling approach is that the dynamic interactions of complex coastal processes occurring in a region are overly simplified into the geometric mean of seven variables and exposure categories (Arkema et al., 2017; Sharp et al., 2018). InVEST does not model storm surge or wave fields in nearshore regions. More importantly, the model does not consider the amount and quality of habitats, and it does not provide quantitative estimates of how habitats reduce coastal hazards. The model does not consider any hydrodynamic or sediment transport processes: it assumes that regions that belong to the same broad geomorphic exposure class behave similarly. In addition, using this model, we assume that natural habitats provide protection to regions that are protected against erosion independent of their geomorphology classification (i.e., rocky cliffs). This limitation artificially deflates the relative vulnerability of these regions and inflates the relative vulnerability of regions that have a high geomorphic index. Based on these limitations and assumptions, the InVEST Coastal Vulnerability tool is an informative approach to investigate relative exposure for a coastline and identify locations where coastal habitats contribute significantly to reducing exposure (Arkema et al., 2013; Ruckelshaus et al., 2016). However, for local scale decisions regarding locally specific geomorphic conditions, more quantitative analysis including local nearshore processes is needed.

**Coastal Zoning Maps**

We aimed to identify where areas of high exposure to coastal hazards (high Exposure Index) aligned with areas of relatively dense residential and commercial development, particularly in areas of key economic or cultural significance for Marin County. The Coastal Vulnerability Model produced a shapefile containing the Exposure Index value for each segment of coast. We combined this with zoning maps (Marin County Community Development Agency, 2015) to locate where various adaptation options would be more feasible. The zone types included:

- Agriculture Production Zone
- Agriculture Residential Planned
- Agriculture and Conservation
- Limited Roadside Business
- Open Area
- Residential Agriculture
- Residential Multiple Planned
- Residential Single Family
- Residential Single Family Planned
- Residential Two Family
- Resort and Commercial Recreation
- Village Commercial Residential

Where high Exposure Index coincided with more dense development types (Residential Two Family, Resort and Commercial Recreation, Village Commercial Recreation), we considered adaptation strategies involving “retreat” options to be more difficult. Conversely, in spaces designated as Agriculture, Open Area, or any “Planned” development (not yet developed), we considered nature-based strategies and preventative policies to be more feasible.

**References**

Arkema, K.K., Griffin, R., Maldonado, S., Silver, J., Suckale, J., Guerry, A.D., 2017. Linking social, ecological, and physical science to advance natural and nature-based protection for coastal communities: Advancing protection for coastal communities. Annals of the New York Academy of Sciences 1399, 5–26. https://doi.org/10.1111/nyas.13322

Arkema, K.K., Guannel, G., Verutes, G., Wood, S.A., Guerry, A., Ruckelshaus, M., Kareiva, P., Lacayo, M., Silver, J.M., 2013. Coastal habitats shield people and property from sea-level rise and storms. Nature Climate Change 3, 913–918. https://doi.org/10.1038/nclimate1944

California Coastal Commission, 2014. Coastal Erosion Armoring (Spatial Dataset). Coastal Sediment Master Plan Spatial Data Database, San Francisco, California. URL http://www.dbw.ca.gov/csmw/SpatialData.aspx

California Department of Fish and Wildlife, 2015. Marine Region GIS Downloads [WWW Document]. Marine Regions GIS Downloads. URL https://www.wildlife.ca.gov/Conservation/Marine/GIS/Downloads (accessed 11.20.18).

Foxgrover, A., Barnard, P., 2012. A Seamless, High-Resolution Digital Elevation Model (DEM) of the North-Central California Coast (Data Series). United States Geological Survey, Reston, VA.

Kraus, N.C., 1988. The Effects of Seawalls on the Beach: An Extended Literature Review. Journal of Coastal Research 4, 1–28.

Marin County Community Development Agency, 2015. Marine County Zoning.

National Oceanic and Atmospheric Administration, Office of Response and Restoration, 2002. Environmental Sensitivity Index Guidelines, Version 3.0 (NOAA Technical Memorandum NOS OR&R 11). Hazardous Materials Response Division, Seattle, WA.

National Research Council, 2012. Sea-Level Rise for the Coasts of California, Oregon, and Washington: Past, Present, and Future. National Academies Press, Washington, D.C. https://doi.org/10.17226/13389

Ruckelshaus, M.H., Guannel, G., Arkema, K., Verutes, G., Griffin, R., Guerry, A., Silver, J., Faries, J., Brenner, J., Rosenthal, A., 2016. Evaluating the Benefits of Green Infrastructure for Coastal Areas: Location, Location, Location. Coastal Management 44, 504–516. https://doi.org/10.1080/08920753.2016.1208882

Sharp, R., Tallis, H., Ricketts, T., Guerry, A.D., Wood, S.A., Chaplin-Kramer, R., Nelson, E., Ennaanay, D., Wolny, S., Olwero, N., Vigerstol, K., Pennington, D., Mendoza, G., Aukema, J., Foster, J., Forrest, J., Cameron, D., Arkema, K., Lonsdorf, E., Kenedy, C., Verutes, G., Kim, C.K., Guannel, G., Papenfus, M., Toft, J., Marsik, M., Berhardt, J., Griffin, R., Glowinski, K., Chaumont, N., Perelman, A., Lacayo, M., Mandle, L., Hamel, P., Vogl, A., Rogers, L., Bierbower, W., 2018. InVEST User’s Guide. The Natural Capital Project, Stanford University, University of Minnesota, The Nature Conservancy, and World Wildlife Fund, Stanford, California.

Tolman, H.L., National Oceanic and Atmospheric Administration, 2014. User manual and system documentation of WAVEWATCH III R version 4.18 (Technical Report). Environmental Modeling Center, College Park, MD.
